# hCTLA4-Gene-Modified Human Bone Marrow-Derived Mesenchymal Stem Cells (hBMMSCs) Maintain POSTN Secretion to Enhance the Migration Capability of Allogeneic hBMMSCs through the Integrin *α*v*β*3/FAK/ERK Signaling Pathway

**DOI:** 10.1155/2020/3608284

**Published:** 2020-03-24

**Authors:** Lei Song, Fei Zhang, Rui Zhou, Jun Xiao, Lei He, Fei Dai

**Affiliations:** ^1^Department of Orthopaedics, First Affiliated Hospital, Army Medical University, Chongqing 400038, China; ^2^Department of Traumatic Orthopaedics, General Hospital of Xinjiang Military Region, Urumqi 830000, China

## Abstract

Cytotoxic T-lymphocyte-associated protein 4- (CTLA4-) modified human bone marrow-derived mesenchymal stem cells (hBMMSCs) might be promising seed cells for bone tissue engineering. However, the underlying mechanism is not clear. In the present study, we investigated whether CTLA4-modified hBMMSCs are involved in the migration of allogeneic hBMMSCs (allo-hBMMSCs) by maintaining POSTN secretion. hBMMSCs were isolated from different groups, named hBMMSCs and allo-hBMMSCs. hBMMSCs that were infected with the negative control (NC), empty adenovirus- or recombinant adenovirus-expressing CTLA4, POSTN, or CTLA4 plus the shRNA of POSTN were named NC hBMMSCs, CTLA4-modified hBMMSCs, POSTN-modified hBMMSCs, or CTLA4+shPOSTN-modified hBMMSCs, respectively. They were then cocultured with PBMCs in a 1 : 5 ratio with 2.5 *μ*g/mL phytohemagglutinin (PHA). The coculture supernatant was collected to treat allo-hBMMSCs with anti-integrin *α*v*β*3 IgG, or negative control IgG, as a control. Following this, ELISA, Transwell assays, wound healing assays, and western blotting were performed. We found that the POSTN level was higher in the culture supernatant of CTLA4- and POSTN-modified hBMMSCs than in NC hBMMSCs cocultured with PBMCs treated with PHA. The migration capability of allo-hBMMSCs was enhanced, and the integrin *α*v*β*3/FAK/ERK signaling pathway in allo-hBMMSCs was activated by the culture supernatant of CTLA4- and POSTN-modified hBMMSCs cocultured with PBMCs treated with PHA. Additionally, these induced effects can be weakened by POSTN knockdown, and the migration capability of allo-hBMMSCs was blocked by anti-integrin *α*v*β*3 IgG. In conclusion, hCTLA4-gene-modified hBMMSCs maintain POSTN secretion to enhance the migration capability of allogeneic hBMMSCs through the integrin *α*v*β*3/FAK/ERK signaling pathway in the T cell immune activation environment.

## 1. Introduction

Large bone defects, caused by fracture, tumor resection, and bone infection, are very common in orthopedic clinical practice. Recently, the tissue-engineered bone (TEB) developed rapidly and achieved a favorable curative effect in repairing large bone defects [1]. Human bone marrow mesenchymal stem cells (hBMMSCs) are important seed cells in TEB [2, 3]. However, autogenous MSCs are limited by patients' individual differences and their finite number; thus, autogenous MSCs cannot meet the needs of clinical treatment and TEB industrialization. Allogeneic MSCs may therefore be another seed cell source for bone tissue engineering, due to their unlimited number. Although the immunogenicity of allogeneic MSCs is low, it cannot be completely disregarded, as it can still induce immune excretion in allogeneic transplantation [4]. Allogeneic hBMMSC transplantation almost invariably results in transplant rejection. Hence, new ways to overcome transplant rejection along with allotransplantation are urgently required.

The immunosuppressive molecule cytotoxic T-lymphocyte-associated protein 4 (CTLA4), a well-known ligand for CD80/CD86, is a coinhibitory molecule expressed on T cells that mediates the inhibition of T cell function. In our previous study, we found that CTLA4 enhanced the osteogenic differentiation of allogeneic hBMMSCs in a model of T cell immune activation *in vitro* [5]. In addition, we observed that CTLA4 is favorable for hBMMSC engraftment and osteogenic differentiation in allogeneic recipients [6]. These results suggested that CTLA4-modified hBMMSCs might be promising seed cells for bone tissue engineering. However, the mechanism underlying this process is not clear.

Importantly, we found that the majority of cells on decalcified bone matrix seeded with CTLA4-modified hBMMSCs were derived from the host itself at two, four, eight, and twelve weeks after transplantation [6]. We therefore predicted that CTLA4-modified hBMMSCs may enhance the recruitment of host MSCs, consequently promoting the repair of bone defects in allogeneic transplantation. Another recent study of ours suggested that CTLA4 promotes MSC osteogenesis in xenotransplantation under T cell immune activation conditions by maintaining periostin (POSTN) expression [7]. POSTN, also known as osteoblast specific factor 2 (OSF-2), is a noncollagenous extracellular matrix (ECM) protein secreted by osteoblasts and their progenitors [8]. POSTN contributes to a microenvironment of bone tissue together with collagen fibers, fiber-junction proteins, hyaline proteins, lamellar bonding proteins, bone bonding proteins, and osteopontin [9, 10]. ECM plays an important role in cellular communication [11, 12]. Moreover, it is reported that POSTN could promote the migration and osteogenic differentiation potential of periodontal ligament MSCs [13]. Therefore, we predicted that CTLA4-modified hBMMSCs are involved in the recruitment of host MSCs by increasing the secretion of POSTN.

In the present study, we investigated whether CTLA4-modified hBMMSCs are involved in the migration of allogeneic hBMMSCs by maintaining POSTN secretion in the T cell immune activation microenvironment *in vitro.* Additionally, we elucidated the underlying mechanism.

## 2. Materials and Methods

### 2.1. Isolation and Identification of hBMMSCs

All enrolled volunteers who provided bone marrow were randomly divided into two groups (*n* = 3; all males; age range: 25-28 years old). A written informed consent was obtained from all volunteers. This study was approved by the ethics committee of the First Affiliated Hospital of Army Medical University. hBMMSCs were isolated as per our previously described method [5, 6]. Bone marrow aspirates from the iliac crest from both groups were separately used to isolate MSCs. The MSCs isolated from group one were named hBMMSCs and were infected with adenovirus-expressing CTLA4, POSTN, or shRNA-targeting POSTN (shPOSTN). The hBMMSCs isolated from the other group were named allogeneic hBMMSCs (allo-hBMMSCs). Isolated hBMMSCs and allo-hBMMSCs were mixed in a 1 : 1 ratio and identified using stem cell positive markers CD105, CD44, and CD90 and stem cell negative markers CD34 and CD45, by flow cytometry.

### 2.2. Isolation of Peripheral Blood Mononuclear Cells (PBMCs)

The heparinized peripheral blood was collected from healthy human volunteers. PBMCs were isolated by the Ficoll density gradient centrifugation from the peripheral blood. The isolated PBMCs were cultured in the RPMI 1640 medium (Gibco, Carlsbad, CA, USA) with 10% fetal bovine serum (FBS) (Gibco), supplemented with 100 U/mL penicillin, 100 U/mL streptomycin, and 2 mM glutamine.

### 2.3. Establishment of Experimental Models

To establish the T cell immune activation microenvironment, the PBMCs (5 × 10^5^ cells) were stimulated with 2.5 *μ*g/mL phytohemagglutinin (PHA) (Sigma, St. Louis, MO, USA), an agent used for polyclonal T cell stimulation [14]. After being treated with or without PHA for 24, 48, 72, and 96 h, the culture medium was collected to detect the levels of interleukin- (IL-) 2 and interferon- (IFN-) *γ*. The levels of IL-2 and IFN-*γ* were measured using the ELISA kit (R&D Systems, Minneapolis, MN, USA).

Recombinant adenovirus-expressing CTLA4, POSTN, or the shRNA of POSTN were purchased from GenePharma (Shanghai GenePharma Co., Ltd., Shanghai, China). hBMMSCs infected with the negative control (NC), empty adenovirus- or recombinant adenovirus-expressing CTLA4, POSTN, or the shRNA of POSTN were named NC hBMMSCs, CTLA4-modified hBMMSCs, POSTN-modified hBMMSCs, or CTLA4+shPOSTN-modified hBMMSCs, respectively.

NC hBMMSCs, CTLA4-modified hBMMSCs, POSTN-modified hBMMSCs, or CTLA4+shPOSTN-modified hBMMSCs (1 × 10^5^ cells) were cocultured with PBMCs (5 × 10^5^ cells) at a ratio of 1 : 5 with 2.5 *μ*g/mL PHA. After treatment with PHA for 72 h, the culture supernatant was collected to detect the levels of POSTN using the ELISA kit (R&D Systems). The culture supernatant was concentrated at a ratio of 10 : 1 for the following assays.

To investigate the effect of NC hBMMSCs, CTLA4-modified hBMMSCs, POSTN-modified hBMMSCs, or CTLA4+shPOSTN-modified hBMMSCs on the migration of allo-hBMMSCs, the concentrated culture supernatant from these cells cocultured with PBMCs stimulated with PHA was added into the culture medium of allo-hBMMSCs at a ratio of 1 : 10, and allo-hBMMSCs were named as NC, CTLA4, POSTN, and CTLA4+shPOSTN, respectively.

To investigate whether CTLA4 or POSTN affects the migration of allo-hBMMSCs via integrin *α*v*β*3, 2 *μ*g/mL negative control IgG or anti-integrin *α*v*β*3 IgG was added into the culture medium of allo-hBMMSCs with the concentrated culture supernatant from CTLA4-modified hBMMSCs or POSTN-modified hBMMSCs cocultured with PBMCs stimulated with PHA, and allo-hBMMSCs were named as CTLA4+IgG, CTLA4+anti-integrin *α*v*β*3, POSTN+IgG, and POSTN+anti-integrin *α*v*β*3, respectively.

### 2.4. Transwell Migration Assay

Totally, 10,000 allo-hBMMSCs were added into the upper portion of a Transwell chamber (24 wells, 8.0 *μ*m). A culture medium (600 *μ*L) containing 20% FBS was added into the lower chamber. After culturing in different conditions for 24 h, the upper-surface allo-hBMMSCs were wiped off with cotton balls, and the allo-hBMMSCs that migrated to the reverse side of the Transwell membrane were washed with PBS, fixed with 5% glutaraldehyde at 4°C, and stained with crystal violet (0.5%) stain solution for 5-10 min. After washing with PBS twice, five fields were randomly observed under the microscope to count the number of migrated allo-hBMMSCs per field.

### 2.5. Wound Healing Assay

After drawing five straight lines on the bottom of a 6-well plate, 1 × 10^6^ cells/well allo-hBMMSCs were seeded. Next day, a wound was generated along the five straight lines using a sterile 200 *μ*L pipette tip. Allo-hBMMSCs were washed with PBS three times to remove detached cells. Subsequently, a 2.5 mL serum-free medium containing a 10% concentrated culture supernatant from MSCs, MSCs-CTLA4, MSCs-POSTN, or MSCs-CTLA4-ShPOSTN groups, or a concentrated culture supernatant from CTLA4-modified hBMMSCs or POSTN-modified hBMMSCs with 2 *μ*g/mL negative control IgG or integrin *α*v*β*3, was added and the cells were cultured at 37°C in a humidified atmosphere with 5% CO_2_. Wound width was monitored and photographed at 48 h using a phase-contrast microscope (Motic Microscopy, Xiamen, China). The percentage of wound closure was calculated using the following formula

(1) $$\begin{array}{c} \text{Percentage of wound closure}=\frac{\text{Wound width at }0\text{ h}}{\text{Wound width at }48\text{ h}}\times 100\%. \end{array}$$

### 2.6. Western Blot

Totally, 1 × 10^6^ allo-hBMMSCs per well were seeded in a 6-well plate and cultured in different conditions. After culturing for 24 h, the cells were collected for western blot to detect the expression levels of MMP2, MMP9, integrin *α*v*β*3, FAK, p-FAK, ERK1/2, p-ERK1/2, AKT1/2, and p-AKT1/2. Western blot was carried out as previously described [5].

### 2.7. Statistical Analysis

The results of three independent experiments are shown as means ± standard deviation. Statistical analysis was performed using the SPSS software version 19.0 (IBM, Chicago, IL, USA). Statistical analysis was carried out using one-way ANOVA followed by the post hoc LSD test. A value of *P* < 0.05 was considered statistically significant.

## 3. Results

### 3.1. Cell Models Were Successfully Constructed

**Figure 1:**
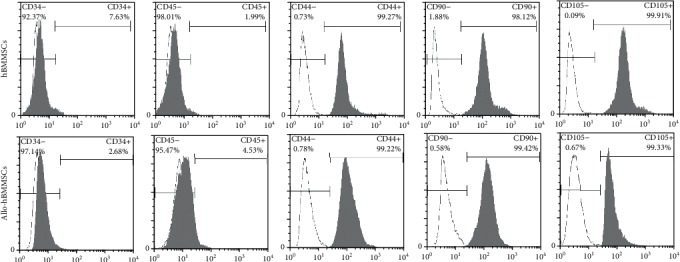
hBMMSCs and allo-hBMMSCs were successfully isolated. Cell proportions of stem cell positive markers (CD44, CD105, and CD90) and stem cell negative markers (CD34 and CD45) were identified by the flow cytometric analysis.

For hBMMSCs, cell proportions of stem cell positive markers CD44, CD105, and CD90 were 99.27%, 98.12%, and 99.91%, respectively, and the cell proportions of stem cell negative markers CD34 and CD45 were 7.63% and 1.99%, respectively (Figure 1). For allo-hBMMSCs, the cell proportions of stem cell positive markers CD44, CD105, and CD90 were 99.22%, 99.42%, and 99.33%, whereas those of stem cell negative markers CD34 and CD45 were 2.68% and 4.53%, respectively (Figure 1). These results indicated that hBMMSCs and allo-hBMMSCs were successfully isolated.

**Figure 2:**
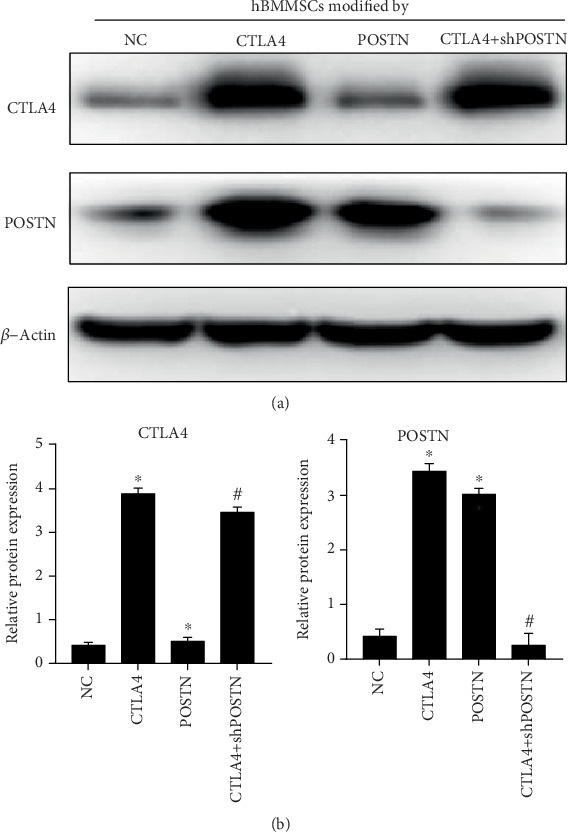
CTLA4 and POSTN levels in NC hBMMSCs, CTLA4-modified hBMMSCs, POSTN-modified hBMMSCs, or CTLA4+shPOSTN-modified hBMMSCs. (a) Representative graphs of western blotting. (b) Statistical results of relative protein expression based on three independent experiments. ^∗^*P* < 0.05, when compared to NC hBMMSCs; ^#^*P* < 0.05, when compared to POSTN-modified hBMMSCs.

As shown in Figure 2, CTLA4 was successfully overexpressed in CTLA4-modified hBMMSCs and CTLA4+shPOSTN-modified hBMMSCs, and POSTN was successfully overexpressed in CTLA4-modified hBMMSCs and POSTN-modified hBMMSCs. The POSTN level in CTLA4+shPOSTN-modified hBMMSCs was less than that in CTLA4-modified hBMMSCs and had no obvious change compared to NC hBMMSCs, indicating that shPOSTN successfully silenced CTLA4-induced POSTN upregulation.

**Figure 3:**
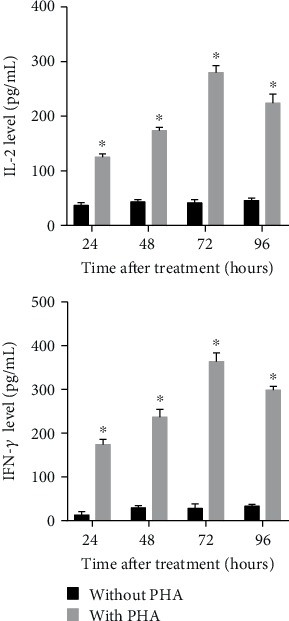
Levels of IL-2 and IFN-*γ* in the culture supernatant of PBMCs treated with PHA. After treatment with or without PHA for 24, 48, 72, and 96 h, IL-2 and IFN-*γ* levels were detected by ELISA. ELISA was performed using the culture supernatant isolated from three independent cell treatments. ^∗^*P* < 0.05.

As shown in Figure 3, the levels of IL-2 and IFN-*γ* in the culture supernatant of PBMCs increased after being treated with PHA for 24, 48, 72, and 96 h, and IL-2 and IFN-*γ* levels reached their peak at 72 h. Therefore, the conditions under which PBMCs were treated with PHA for 72 h were chosen as the appropriate time to represent the T cell immune activation microenvironment.

**Figure 4:**
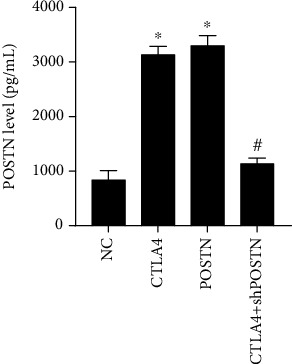
POSTN levels in the coculture supernatant. NC hBMMSCs, CTLA4-modified hBMMSCs, POSTN-modified hBMMSCs, or CTLA4+shPOSTN-modified hBMMSCs were cocultured with PBMCs treated with PHA for 72 h, and the coculture supernatant was isolated to detect the POSTN level by ELISA. ELISA was performed using the coculture supernatant isolated from three independent cell treatments. ^∗^*P* < 0.05, when compared to NC group; ^#^*P* < 0.05, when compared to the POSTN group.

As shown in Figure 4, the POSTN level was higher in the culture supernatant of CTLA4- and POSTN-modified hBMMSCs than in that of NC hBMMSC cocultured with PBMCs treated with PHA. In addition, POSTN levels in the culture supernatant of CTLA4+shPOSTN-modified hBMMSCs were lower than those in the culture supernatant of CTLA4-modified hBMMSCs and had no obvious change compared to the culture supernatant of NC hBMMSCs, indicating that shPOSTN successfully inhibits the secretion of POSTN maintained by CTLA4 in CTLA4-modified hBMMSCs cocultured with PBMCs treated with PHA.

All the above results indicated that the overexpression of POSTN in the culture supernatant of CTLA4- or POSTN-modified hBMMSCs can be maintained in a T cell immune activation microenvironment, and that these cell models are suitable for investigating whether CTLA4-modified hBMMSCs are involved in the migration of allogeneic hBMMSCs by promoting POSTN secretion in a T cell immune activation microenvironment *in vitro*.

### 3.2. Migration Capability of Allo-hBMMSCs Was Enhanced by the Culture Supernatant of CTLA4- and POSTN-Modified hBMMSCs Cocultured with PBMCs Treated with PHA

**Figure 5:**
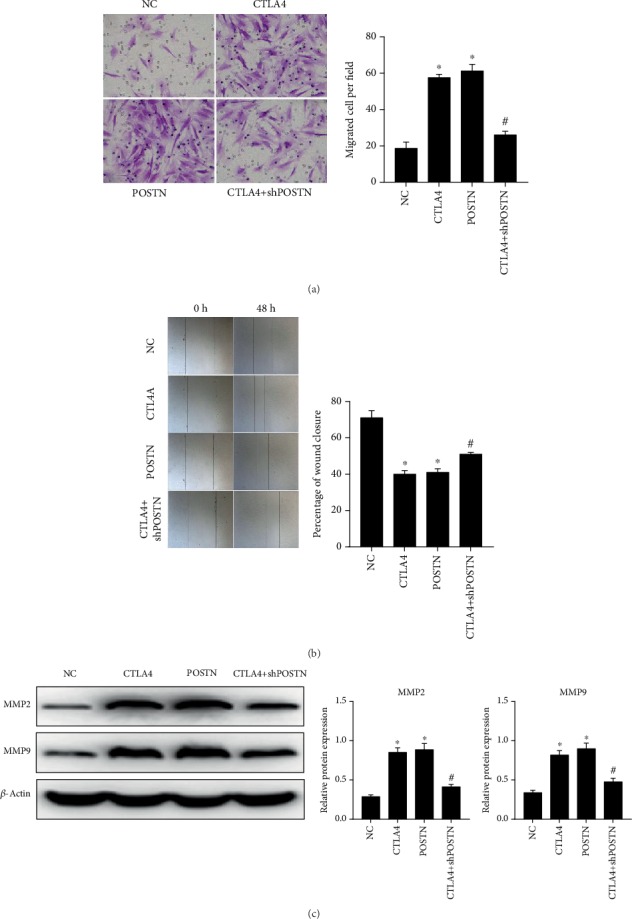
Migration capability of allo-hBMMSCs was enhanced by the culture supernatant of CTLA4- and POSTN-modified hBMMSCs cocultured with PBMCs treated with PHA. The concentrated culture supernatant from NC hBMMSCs, CTLA4-modified hBMMSCs, POSTN-modified hBMMSCs, or CTLA4+shPOSTN-modified hBMMSCs cocultured with PBMCs treated with PHA was added into the culture medium of allo-hBMMSCs in a ratio of 1 : 10, and the treated allo-hBMMSCs were named as NC, CTLA4, POSTN, and CTLA4+shPOSTN, according to their treatment. The migration capability of allo-hBMMSCs in each group was evaluated by Transwell assay (a), wound healing assay (b), and western blot to detect MMP2 and MMP9 levels (c). For all panels, left is the representative graphs and right is the statistical results based on three independent experiments. ^∗^*P* < 0.05, when compared to the NC group; ^#^*P* < 0.05, when compared to the POSTN group.

The results of the Transwell assay showed that the number of migrated allo-hBMMSCs in CTLA4 and POSTN groups was more than that in the NC group, and the number of migrated allo-hBMMSCs in the CTLA4+shPOSTN group was less than that in the CTLA4 group (Figure 5(a)). The percentage of wound closure of allo-hBMMSCs in CTLA4 and POSTN groups was higher than that in the NC group, and the percentage of wound closure of allo-hBMMSCs in the CTLA4+shPOSTN group was lower than that in the CTLA4 group (Figure 5(b)). Moreover, MMP2 and MMP9 expression levels in allo-hBMMSCs in CTLA4 and POSTN groups were higher than those in the NC group, and MMP2 and MMP9 expression levels in allo-hBMMSCs in the CTLA4+shPOSTN group were lower than those in the CTLA4 group (Figure 5(c)).

### 3.3. Integrin *α*v*β*3/FAK/ERK Signaling Pathway in Allo-hBMMSCs Was Activated by the Culture Supernatant of CTLA4- and POSTN-Modified hBMMSCs Cocultured with PBMCs Treated with PHA

**Figure 6:**
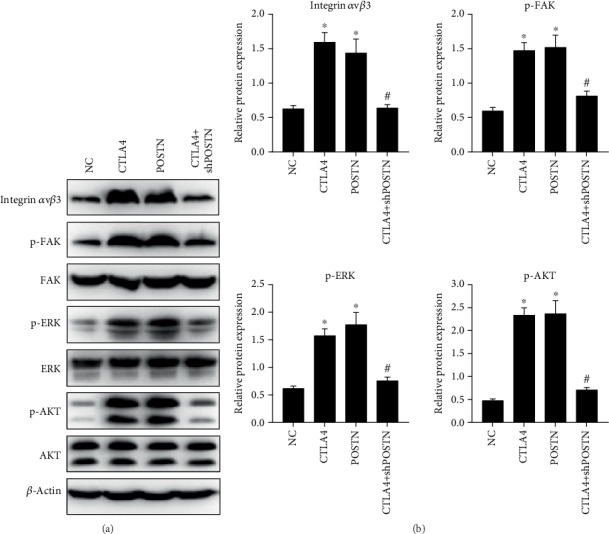
The integrin *α*v*β*3/FAK/ERK signaling pathway in allo-hBMMSCs was activated by the culture supernatant of CTLA4- and POSTN-modified hBMMSCs cocultured with PBMCs treated with PHA. The concentrated culture supernatant from NC hBMMSCs, CTLA4-modified hBMMSCs, POSTN-modified hBMMSCs, or CTLA4+shPOSTN-modified hBMMSCs cocultured with PBMCs treated with PHA was added into the culture medium of allo-hBMMSCs at a ratio of 1 : 10, and the treated allo-hBMMSCs were named NC, CTLA4, POSTN, and CTLA4+shPOSTN, according to their treatment. Protein levels of key factors in the integrin *α*v*β*3/FAK/ERK signaling pathway were detected by western blotting. (a) Representative graphs of western blotting and (b) statistical results of relative protein expression based on three independent experiments. ^∗^*P* < 0.05, when compared to NC group; ^#^*P* < 0.05, when compared to the POSTN group.

Integrin *α*v/*β*3, p-FAK, p-ERK1/2, and p-AKT1/2 expression levels in allo-hBMMSCs in the CTLA4 and POSTN groups were higher than those in the NC group, whereas the total FAK, ERK1/2, and AKT1/2 expression levels did not exhibit a considerable change (Figure 6). In addition, integrin *α*v*β*3, p-FAK, p-ERK1/2, and p-AKT1/2 expression levels in allo-hBMMSCs in the CTLA4+shPOSTN group were lower than those in the CTLA4 group, whereas the total FAK, ERK1/2, and AKT1/2 expression levels did not show a substantial change (Figure 6). These results indicate that the integrin *α*v*β*3/FAK/ERK signaling pathway was activated by the culture supernatant of CTLA4- and POSTN-modified hBMMSCs cocultured with PBMCs treated with PHA, and the activation induced by CTLA4 can be suppressed by shPOSTN.

### 3.4. Anti-Integrin *α*v*β*3 Blocked the Effect of the Culture Supernatant of CTLA4- and POSTN-Modified hBMMSCs Cocultured with PBMCs Treated with PHA

**Figure 7:**
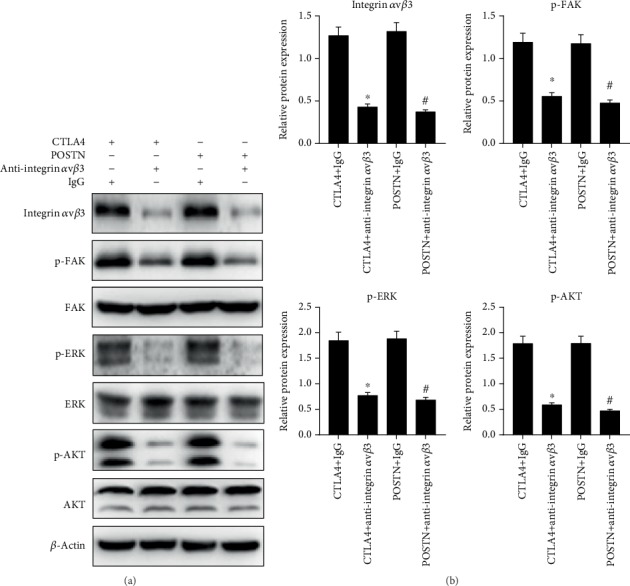
Integrin *α*v*β*3/FAK/ERK signaling pathway was blocked by anti-integrin *α*v*β*3 treatment. Approximately 2 *μ*g/mL negative control IgG or anti-integrin *α*v*β*3 was added into the culture medium of allo-hBMMSCs with the concentrated culture supernatant from CTLA4-modified hBMMSCs or POSTN-modified hBMMSCs and allo-hBMMSCs cocultured with PBMCs treated with PHA, and treated allo-hBMMSCs were named CTLA4+IgG, CTLA4+anti-integrin *α*v*β*3, POSTN+IgG, or POSTN+anti-integrin *α*v*β*3. Protein levels of key factors in the integrin *α*v*β*3/FAK/ERK signaling pathway were detected by western blotting. (a) Representative graphs of western blotting and (b) statistical results of relative protein expression based on three independent experiments. ^∗^*P* < 0.05, when compared to the IgG group.

Integrin *α*v/*β*3, p-FAK, p-ERK1/2, and p-AKT1/2 expression levels in allo-hBMMSCs in CTLA4+anti-integrin *α*v*β*3 and POSTN+anti-integrin *α*v*β*3 groups were lower than those in the CTLA4+IgG and POSTN+IgG groups, whereas the total FAK, ERK1/2, and AKT1/2 expression levels did not show a considerable change (Figure 7). These results indicate that anti-integrin *α*v*β*3 blocked the effect of the culture supernatant of CTLA4- and POSTN-modified hBMMSCs cocultured with PBMCs treated with PHA.

**Figure 8:**
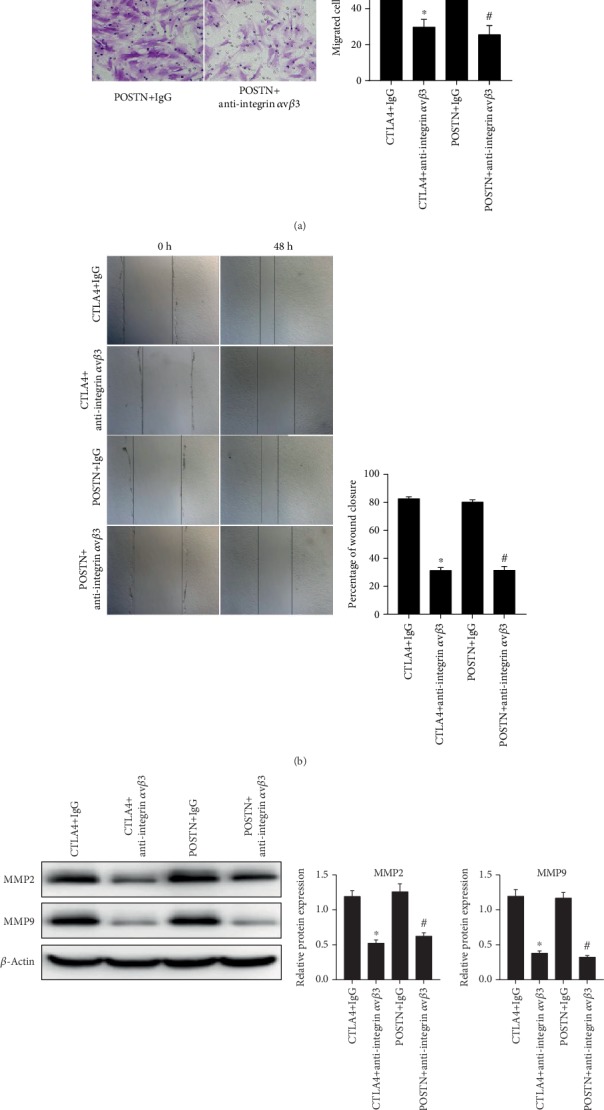
Migration capability of allo-hBMMSCs in each group was suppressed by anti-integrin *α*v*β*3 treatment. Approximately 2 *μ*g/mL negative control IgG or anti-integrin *α*v*β*3 was added to the culture medium of allo-hBMMSCs with concentrated culture supernatant from CTLA4-modified hBMMSCs or POSTN-modified hBMMSCs and allo-hBMMSCs co-cultured with PBMCs treated with PHA, and the treated allo-hBMMSCs were named CTLA4+IgG, CTLA4+anti-integrin *α*v*β*3, POSTN+IgG, or POSTN+anti-integrin *α*v*β*3. The migration capability of allo-hBMMSCs in each group was evaluated by Transwell assay (a), wound healing assay (b), and western blotting to detect MMP2 and MMP9 levels (c). For all panels, left shows the representative images or blots and right shows the statistical results based on three independent experiments. ^∗^*P* < 0.05, when compared to the IgG group.

The results of the Transwell assay showed that the numbers of migrated allo-hBMMSCs in the CTLA4+anti-integrin *α*v*β*3 and the POSTN+anti-integrin *α*v*β*3 groups were less than those in CTLA4+IgG and POSTN+IgG groups (Figure 8(a)). The percentages of wound closure of allo-hBMMSCs in CTLA4+anti-integrin *α*v*β*3 and POSTN+anti-integrin *α*v*β*3 groups were lower than those in CTLA4+IgG and POSTN+IgG groups (Figure 8(b)). Moreover, MMP2 and MMP9 expression levels in allo-hBMMSCs in the CTLA4+anti-integrin *α*v*β*3 and POSTN+anti-integrin *α*v*β*3 groups were lower than those in the CTLA4+IgG and POSTN+IgG groups (Figure 8(c)).

## 4. Discussion

hBMMSCs are important seed cells in TEB [2, 3]. Alongside autogenous hBMMSCs, allo-hBMMSCs are also a crucial seed cell source in TEB because of their unlimited number. However, transplantation rejection induced by allogeneic hBMMSC transplantation limits its application in the clinic. Our previous study showed that CTLA4-modified hBMMSCs could keep the osteogenic differentiation in the T cell immune activation microenvironment *in vivo* [5], indicating that CTLA4-modified hBMMSCs may be a promising source for TEB. Thus, it is critical to further evaluate their potential as a seed cell source for CTLA4-modified hBMMSCs in TEB to overcome transplantation rejection. Our study suggested that CTLA4-modified hBMMSCs may enhance the recruitment of host MSCs in a certain way [6]. We therefore predicted that CTLA4-modified hBMMSCs are involved in the migration of allogeneic hBMMSCs. Besides testing this hypothesis, we also discussed the underlying mechanism.

To investigate the effect of CTLA4-modified hBMMSCs on the migratory capability of allo-hBMMSCs in a T cell immune activation microenvironment, CTLA4-modified hBMMSCs were cocultured with PBMCs treated with PHA to simulate the immune activation microenvironment, and the coculture medium was collected to treat allo-hBMMSCs. Our results showed that the number of migrated allo-hBMMSCs in the CTLA4 and POSTN groups was more than that in the NC group, and the percentage of wound closure of allo-hBMMSCs in the CTLA4 and POSTN groups was higher than that in the NC group. In addition, MMP2 and MMP9 expression levels in allo-hBMMSCs in the CTLA4 and POSTN groups were higher than those in the NC group. MMP2 and MMP9 are important proteins that promote cell migration [15, 16]. Taken together, our results indicate that the culture supernatant from CTLA4-modified hBMMSCs could enhance the migratory capability of allo-hBMMSCs in a T cell immune activation microenvironment. This is consistent with our previous *in vivo* study wherein the majority of cells on decalcified bone matrix seeded with CTLA4-modified hBMMSCs were derived from the host itself at two, four, eight, and twelve weeks after transplantation [6].

We found that the culture supernatants of POSTN-modified hBMMSCs and CTLA4-modified hBMMSCs have similar effects on the migratory capability of allo-hBMMSCs in a T cell immune activation microenvironment. In addition, the number of migrated allo-hBMMSCs, the percentage of wound closure of allo-hBMMSCs, and MMP2 and MMP9 expression levels in allo-hBMMSCs in the CTLA4+shPOSTN group were lower than those of the CTLA4 group, indicating that POSTN knockdown weakened the effects induced by the culture supernatant of CTLA4-modified hBMMSCs cocultured with PBMCs treated with PHA. Our present results support the prediction that CTLA4-modified hBMMSCs enhanced the migratory capability of allo-hBMMSCs by maintaining the secretion of POSTN in a T cell immune activation microenvironment. This may be the mechanism underlying the CTLA4-modified hBMMSC involvement in the recruitment of host MSCs [6]. Our results can be supported by the role of POSTN on migration in MSCs. It is reported that recombinant POSTN protein stimulates the migration of human adipose tissue-derived MSCs *in vitro* [17].

To fully elucidate the mechanism, an important question should be solved: how does POSTN secretion by CTLA4-modified hBMMSCs affect the migratory capability of allo-hBMMSCs? It has been reported that POSTN regulates MMP-2 expression via activation of the *α*v*β*3 integrin/ERK pathway expression in human periodontal ligament cells [18]. In addition, POSTN produced by human periodontal ligament fibroblasts promotes migration of human MSCs through the *α*v*β*3 integrin/FAK/PI3K/Akt pathway [19]. Integrin *α*v*β*3, a member of the integrin family, is a complex of integrin subunit alpha V (CD51) and integrin subunit beta 3 (CD61) [20]. POSTN is an ECM protein, which plays an important role in cellular communication [9, 12]. Based on these reports, we predicted that POSTN secreted by CTLA4-modified hBMMSCs could activate the integrin *α*v*β*3/FAK/ERK signaling pathway of allo-hBMMSCs, resulting in the enhancement of migratory capability of allo-hBMMSCs. We found that the integrin *α*v*β*3/FAK/ERK signaling pathway in allo-hBMMSCs was activated by the culture supernatant of CTLA4- and POSTN-modified hBMMSCs cultured in a T cell immune activation microenvironment, and anti-integrin *α*v*β*3 IgG blocked the effect of the culture supernatant of CTLA4- and POSTN-modified cultured hBMMSCs on the migratory capability of allo-hBMMSCs in a T cell immune activation microenvironment. Our present results therefore support the above prediction.

## 5. Conclusions

hCTLA4-gene-modified hBMMSCs maintain POSTN secretion to enhance the migration capability of allogeneic hBMMSCs through the POSTN/integrin *α*v*β*3/FAK/ERK signaling pathway. Based on this conclusion, we hypothesized that CTLA4-modified hBMMSCs seeded in TEB can recruit host hBMMSCs to the location of bone defects *in vivo*. Because host hBMMSCs can escape from transplantation rejection, recruited host hBMMSCs by CTLA4-modified hBMMSCs in TEB can promote the repair of bone defects. These results further support the use of CTLA4-modified hBMMSCs as seed cells in TEB in the future. However, immune response induced by TEB and seed cells is complex in clinical settings and animal models, not only T cell immune activation. Therefore, there is a question whether hCTLA4-gene-modified hBMMSCs have similar function in clinical settings and animal models. In a further study, we will verify our present results in an entire immune activation environment *in vitro* and animal models.

## Data Availability

The datasets generated and/or analyzed during the current study are available from the corresponding author on reasonable request.

## Abbreviations

TEB: Tissue-engineered bone
hBMMSCs: Human bone marrow mesenchymal stem cells
CTLA4: Cytotoxic T-lymphocyte associated protein 4
POSTN: Periostin
ECM: Extracellular matrix
shPOSTN: shRNA-targeting POSTN
PBMCs: Peripheral blood mononuclear cells
PHA: Phytohemagglutinin
IL-2: Interleukin-2
IFN-*γ*: Interferon-*γ*.
